# Ultrasonic‐assisted extraction of bioactive chlorogenic acid from heilong48 soybean variety: Parametric optimization and evaluation of physicochemical and bioactive properties

**DOI:** 10.1002/fsn3.2670

**Published:** 2022-03-11

**Authors:** Nelson Dzidzorgbe Kwaku Akpabli‐Tsigbe, Yongkun Ma, John‐Nelson Ekumah, Juliet Osabutey, Jie Hu, Manqing Xu, Nana Adwoa Nkuma Johnson, Benjamin Kumah Mintah

**Affiliations:** ^1^ School of Food and Biological Engineering Overseas College of Education Jiangsu University Zhenjiang China; ^2^ Department of Nutrition and Food Science College of Basic and Applied Sciences University of Ghana Legon Ghana; ^3^ Department of Early Childhood Education University of Education Winneba Ghana; ^4^ Virtuous Experimental School Achimota‐Accra Ghana; ^5^ Council for Scientific and Industrial Research (CSIR) Food Research Institute Accra Ghana

**Keywords:** chlorogenic acid, heilong48 soybean variety, optimization, physicochemical properties, ultrasonic‐assisted extraction

## Abstract

Chlorogenic acid (CA), especially that found in soybeans, is a rich bioactive compound but has received very little attention in research settings in past decades. Ultrasonic‐assisted extraction (UAE) could be an efficient method to increase CA release from soybeans. Hence, this study aimed to optimize UAE parameters for CA extraction from heilong48 soybean (HS) variety and evaluate the physicochemical and bioactive properties of the soybean. Optimization of ultrasound parameters with a Box–Behnken design found a frequency of 20.0 kHz, a power density of 30.0 W/L), a temperature of 37.9°C, and a time of 28.0 min to be the best conditions, which gave a CA yield of 5.007 ± 0.033 mg/g and 2,2‐diphenyl‐1‐picrylhydrazyl (DPPH) radical scavenging activity of 93.197 ± 0.213 μmol of AA eq/g dry sample; these were higher than those of a non–ultrasound‐treated (raw) HS sample (CA yield of 1.627 ± 0.528 mg/g and DPPH radical scavenging activity of 10.760 ± 0.207 μmol of AA eq/g dry sample). A satisfactory model was obtained. Scanning electron microscopy results confirmed the structural changes of the HS variety caused by the optimized UAE parameters. High total polyphenol contents (TPCs; 4.726 ± 0.002 mg GAE/g) and total phenolic acids (1.883 ± 0.005 mg GAE/g) and low total flavonoid contents (0.040 ± 0.008 mg RE/g) were obtained. A positive linear correlation between antioxidant activity and TPC was established. A protein–phenolic interaction in the HS variety was observed. The results established that polyphenols should be considered a significant component of the HS variety. Likewise, the HS variety could be used for CA extraction.

## Graphical abstract

A satisfactory ultrasonic‐assisted extraction model was obtained. The heilong48 soybean (HS) variety could be used to produce chlorogenic acid. There were protein–polyphenol interactions in the HS variety.

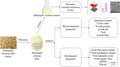

## 1 INTRODUCTION

Soybeans (*Glycine max* L.), from the family Fabaceae, are a good, rich, and vital dietary source of protein, lipids, minerals, vitamins, fiber, and bioactive compounds. Their protein too is rich in essential amino acids (Gorissen et al., 2018; Messina & Messina, 2010). Because of their exceptional nutritional value, soybeans are a vital staple crop in the world. Globally, the role of legumes in traditional diets of various regions is significant. Messina and Messina (2010) showed that soybeans are the only leguminous crop containing high quantities of substances such as alpha‐linolenic acid (Blondeau et al., 2015; Lee et al., 2020) and oleic acid (up to 80%; Lee et al., 2020; Rayaprolu et al., 2015) that are beneficial to humans with respect to preserving the heart and/or blood vessels. Furthermore, significant quantities of polyphenolic compounds are found in soybeans, and this makes them unique among legume crops (Gupta, 2017; Kim et al., 2017; Sakthivelu et al., 2008). Kim et al. (2017) and Kim et al. (2012) found isoflavones and phenolic acids (tocopherols, saponins, and phytic acids) in soybeans. Phenolic acids have lately gained considerable attention in research settings because of their feasible biological effects. Chlorogenic acid (CA) is one of the most significantly available phenolic acids found in soybeans. Chlorogenic acid is an essential bioactive dietary polyphenol that plays important therapeutic roles, including antibacterial, antioxidant, anti‐inflammatory, cardioprotective, neuroprotective, antiviral, antihypertension, antidiabetic, and anti‐obesity roles (Liang & Kitts, 2016; Santana‐Gálvez et al., 2017).

Despite the health/therapeutic advantages of CA, existing literature shows a dearth of information on CA obtained from soybeans, as the majority of available data concentrate on the metabolism and bioactivity of other bioactive compounds found in soybeans (Cao et al., 2019; Křížová et al., 2019). Thus, the majority of these studies dwelled much on substances such as isoflavones from soybeans (Gupta, 2017; Kim et al., 2017; Lee et al., 2020) and soybean meal (Wang et al., 2009) but neglected other bioactive (phenolic) compounds such as CA. Isoflavones are mentioned in the titles or abstracts of more than 17,000 academic articles in the Web of Knowledge (Chen et al., 2012) with no mention of CA. To date, the only literature on soybeans about CA dates back to 1979, and that reports soybeans as a source of CA only without further work/data on content quantification or extraction (Akpabli‐Tsigbe et al., 2021a); this clearly supports the lack of information (knowledge gap) in literature. Recently, a new soybean variety (heilong48) was introduced with improved qualities by Tianxia Agricultural and Sideline Products in China. This variety was developed to have a high phenolic acid value over other soybean varieties. The heilong48 soybean (HS) variety has not been investigated for CA extraction, and its constituents may vary from other varieties and from one producer to another because of differences in the genetic makeup, soil, and environmental conditions. The CA content of the HS variety can be extracted with ultrasound for enhanced yields for food/pharmaceutical applications.

The extraction of several types of plant metabolites that are valuable ingredients to the food, pharmaceutical, and cosmetic industries can be performed with ultrasonic‐assisted extraction (UAE). Alves Filho et al. (2020) studied ultrasound utilization for metabolite extraction from numerous vegetables, fruits, stems, leaves, and agricultural wastes, including blackberries, acerola, olive leaves, sweet potatoes, jabuticaba, and potato peels, among others. Ultrasonic‐assisted extraction is simple, nonthermal, fast, and low cost and hence applicable to the rapid extraction of many phenolic compounds from the plant matrix with no decay into other inactive compounds. However, some researchers have reported on the degradation of anthocyanins by overexposure to the waves and cavitation created by ultrasound (Alves Filho et al., 2020). As a result, the application of UAE needs optimization for each kind of material to be processed; otherwise, phenolic compounds may undergo different degrees of degradation or sonochemical transformation. The correct selection of UAE operating conditions (parameters) makes it a useful method not only for extraction but also for selective extraction of different compounds from herbs, vegetables, and fruits, including purple sweet potato, potato peels, and microalgae (Alves Filho et al., 2020). The present study, therefore, was aimed at optimizing ultrasonic parameters for UAE of CA from the HS variety and evaluating the physicochemical and bioactive characteristics of this soybean variety.

## 2 MATERIALS AND METHODS

Tianxia Agricultural and Sideline Products (China) supplied the HS variety used in the study. Only analytical‐grade chemicals bought from Sinopharm Chemical Reagent Co., Limited (China), were used in this study.

### 2.1 Preparation of flour from the HS variety

Heilong48 soybean flour was prepared according to the method outlined in our previous works (Akpabli‐Tsigbe et al., 2021a, 2021b). Briefly, the HS variety was milled with a hammer crusher (FC160, Shanghai Traditional Chinese Medicine Machinery Factory, China) and further sieved (65‐inch, 0.25‐mm bore diameter; Shaoxing Shangyu Huafeng Hardware Instruments Co., Ltd., China) into fine flour with a particle size of 0.25 mm. The final flour was packed in weights of 150 g in zip‐lock rubber bags and stored (−20°C) for further analyses.

### 2.2 HS extract preparation

The HS extract was prepared according to the method outlined by Ayelign and Sabally (2013) with slight modifications. Two grams of HS flour was accurately weighed into a 250‐ml beaker, and 100 ml of distilled water was added. The solution was boiled for 5 min while being stirred with a magnetic stirrer (model C‐MAG HS 7 S025; IKA, Germany) with a heater set at 95°C (Ullah Shirazi et al., 2014). The solution was allowed to cool and then was filtered through double‐loop qualitative filter paper (No. 1568; Ge Biotechnology Co., Ltd, China) to remove particles. The clear filtrate (extract) obtained was used for total polyphenol content (TPC), total flavonoid content (TFC), total phenolic acid (TPA), and antioxidant activity determination.

### 2.3 UAE of CA

UAE of CA from the HS variety was performed at three frequency levels (20, 40, and 60 kHz) in an ultrasonic water bath (Miebo Biotech Co., China) operated at different power levels (180, 240, and 300 W) of maximum effective ultrasonic power density (30, 40, and 50 W/L, respectively) at different temperature levels (35, 40, and 45°C) for different extraction times (25, 30, and 35 min; Table S1a). Forty milligrams of HS flour was accurately weighed into glass tubes and dissolved with 30 ml of distilled water, and this resulted in a solution of 1.33 mg/ml. Each sample tube was treated with ultrasound in an ultrasonic water bath, and it was positioned above the ultrasonic transducer of the bath to ensure that the ultrasonic intensity for each experimental run was the same. The extracts obtained after sonication were centrifuged for 20 min at 4000 *g* with an RJ‐TDL‐50A centrifuge (Ruijiang Analytical Instrument Corporation Limited, China), filtered, and stored at −20°C for further analyses.

### 2.4 Optimization of ultrasonic parameters for CA extraction using Box–Behnken design (BBD)

Optimization was performed to obtain the maximum yields of CA with improved antioxidant activity via a desirability index (DI). A BBD using response surface methodology was applied to model and optimize sonication parameters and their influences on CA yields and antioxidant activity as responses. The independent parameters used were frequency (A), power density (B), temperature (C), and time (D), which varied from 20 to 60 kHz, from 30 to 50 W/L, from 35 to 45°C, and from 25 to 35 min, respectively. The selection of the levels of the parameters was based on the literature (Alves Filho et al., 2020; Esclapez et al., 2011; Guglielmetti et al., 2017). Box‐Behnken design with four‐factor‐three‐level was used, which gave 29 experimental runs performed on HS extract. Correlation of the relationship of each independent variable to the dependent variable was achieved through the fitting of a second‐degree polynomial model from the following equation:

(1) $$Y=\beta_{0}+\sum_{i=1}^{3}\beta_{i}X_{i}+\sum_{i=1}^{3}\beta_{\mathrm{ii}}X_{i}^{2}+\sum_{i=1}^{3}\times \sum_{j=i+1}^{3}\beta_{\mathrm{ij}}X_{i}X_{j},$$

where *Y* is the predicted response variable, *β*_0_ is the intercept, *β_i_* is the linear regression coefficient of the model, *β_ii_* is the second‐order regression coefficient of the model, *β_ij_* is the estimated interaction regression coefficient of the model, and *X_i_* and *X_j_* are the values of factors. The next equation was used for the selection of the optimized parameters based on the overall DI:

(2) $$\text{DI}={\left[\prod_{i=1}^{3}d_{i}\left(y_{i}\right)\right]}^{\frac{1}{3}},$$

where *d_i_* is the desirability index of the response variable (0–1) and *y_i_* is the responses.

### 2.5 Standard CA solution preparation

The standard CA solution was prepared using the protocol reported by Akpabli‐Tsigbe et al. (2021a). Briefly, 1000 mg was dissolved in 1 L of distilled water to prepare a stock standard CA solution. The solution was thoroughly mixed using a magnetic stirrer (C‐MAG HS 7 S025; IKA, Germany) in the dark. A series of standard solutions (5, 10, 15, 20, 25, and 30 mg/L) were prepared from the stock solution for CA in distilled water. All measurements were performed within 10 min after the preparation, and the absorbance of each series of standard CA solutions was measured immediately. The method was validated against Beer–Lambert's law with the series of standard CA solutions prepared.

### 2.6 Proximate analysis

The moisture content, total solids, fat, protein, crude fiber, and ash were determined with the methods described by AOAC (2005).

The moisture content of HS flour was determined using the hot‐air oven method. A crucible was washed and dried in a GZX‐9240 MBE hot‐air oven (Shanghai Boxun Industrial Co., Ltd., China). The clean, dried, and empty crucible was allowed to cool in a desiccator, weighed and its weight recorded (*W*_1_ [g]). Five grams of HS flour was then weighed into the crucible, and the weight of the crucible with the HS flour sample was recorded (*W*_2_ [g]). The crucible was shaken until the content was evenly distributed and placed in a GZX‐9240 MBE hot‐air oven (Shanghai Boxun Industrial Co., Ltd., China), maintained at a temperature of 105 ± 2°C, and dried for 24 hr. The crucible with the dried sample was cooled in a desiccator, weighed and the weight recorded. It was returned to the oven after weighing, and the process was repeated until a constant weight was obtained (*W*_3_ [g]). The moisture content of the HS sample was calculated with the following formula:

(3) $$\%\text{Moisture}\;\;\text{content}=\frac{W_{2}‐W_{3}}{W_{2}‐W_{1}}\times 100,$$

where *W*_1_ is the weight of the empty crucible, *W*_2_ is the weight of the crucible and sample, and *W*_3_ is the weight of the crucible and sample after drying in the oven.

The total solid by difference was calculated by the subtraction of the percentage of moisture from one hundred (100):

(4) Totalsolids=100‐%Moisturecontent,

The crude fat of HS flour was determined using the Soxhlet apparatus. The extraction flask (a round‐bottom flask) was washed and dried in a GZX‐9240 MBE hot‐air oven (Shanghai Boxun Industrial Co., Ltd., China). The clean, dried, and empty extraction flask was allowed to cool in a desiccator and weighed, and its weight was recorded (*W*_1_ [g]). Two grams of the HS flour sample was transferred into a cleaned, dried, preweighed, and empty extraction thimble (*W*_2_ [g]). The weight of the thimble with the sample was recorded (*W*_3_ [g]). The sample in the thimble was covered/blocked with fat‐free cotton (to prevent the dispersion of the sample in the extraction solvent and siphoning into the extraction flask) and placed into the extractor so that it was within its siphon height. The extraction flask was connected to the extractor carrying the thimble, and a sufficient amount of petroleum ether was poured into the extractor to start the siphon and then filled to about half of the volume of the extraction flask. The extractor was connected to a condenser, and the flask was heated in a water bath for 18 hr. The heat vaporized the solvent and is condensed in the condenser. The condensed solvent fell dropwise into the thimble. The solvent extracted the fat present in the sample. When the level of the solvent reached the siphon height, the whole of the ether flowed down into the flask below and took along the extracted fat. The process was repeated throughout the extraction period. At the end of 18 hr, at which time at least 25–30 siphoning was done, some of the solvents were recovered, and the flask was removed. The flask with the extracted fat was placed in a GZX‐9240 MBE hot‐air oven (Shanghai Boxun Industrial Co., Ltd., China) at 40°C for about 30 min to evaporate off the remaining solvent, and this left only the fat extracted in the flask. The flask was cooled in a desiccator and weighed. The weight was recorded (*W*_4_ [g]). Drying, cooling, and weighing or successive weighing (not reducing by more than 0.0002 g) were performed. The percentage of crude fat (ether extract) extracted from the HS sample was calculated with the following formula:

(5) $$\%\text{Crude}\;\text{fat}=\frac{W_{4}‐W_{1}}{W_{3}‐W_{2}}\times 100,$$

where *W*_1_ is the weight of the empty extraction flask, *W*_2_ is the weight of the empty extraction thimble, *W*_3_ is the weight of the extraction thimble and sample, and *W*_4_ is the weight of the extraction flask and fat after solvent evaporation.

The crude protein of the HS flour was determined by weighing 5 g in cupped filter paper and transferring it into a clean, dry Kjeldahl flask. Ten grams of a digestion mixture (CuSO_4_ + K_2_SO_4_ + selenium oxide) and 25 ml of concentrated H_2_SO_4_ were added. It was slowly warmed to minimize frothing and then boiled until the solution was clear. The solution was allowed to cool and was transferred into a 100‐ml volumetric flask, and the volume was made up with distilled water. Ten milliliters of digest and 10 ml of 40% NaOH were added to the receiver of the distillation apparatus, and this was distilled for half an hour; afterward, the condenser outlet was disconnected. Nitrogen gas released from the distillation was collected in 25 ml of a 4% boric acid solution containing three drops of a mixed indicator (the mixed indicator gave an orange color to the boric acid solution). The orange color of the boric acid solution was changed to a Prussian/sea blue color after nitrogen gas was trapped. The obtained boric acid solution (containing nitrogen gas) was titrated against 0.01 N HCl, and a blank (distilled water) was used in place of the sample. The end point of the titration was indicated by a drop of 0.01 N HCl solution that changed the Prussian/sea blue color of the boric acid solution (containing nitrogen gas) to an orange color. The percentages of nitrogen and crude protein of the HS sample were calculated using Equations (6) and (7), respectively:

Calculation:1mlof0.01NHCltitratedagainstsample=0.0014g,

Titervolume=A‐Bml,

(6) $$\%\text{Nitrogen}=\frac{A‐B\times 0.0014}{W}\times \frac{V}{V_{1}}\times 100,$$

(7) %Crudeprotein=%Nitrogen×Conversionfactor[6.25(Krul,2019)],

where *A* is the volume of 0.01 N HCl titrated against the sample, *B* is the volume of 0.01 N HCl titrated against the blank, *W* is the weight of the sample (5 g), *V* is the total volume of the sample digest (100 ml), and *V*_1_ is the volume of the sample digest distilled (10 ml).

The crude fiber of the HS flour was determined by weighing 2 g of dried, fat‐free residue obtained after crude fat determination, and this was transferred into a 1‐L conical flask. Two hundred milliliters of dilute 1.25% H_2_SO_4_ was boiled in a beaker and poured into the flask. The contents of the flask were boiled for 30 min with constant shaking, and the liquid level was maintained during boiling. At the end of 30 min, the flask was removed, and its contents were filtered through a fine‐lined pad held in a funnel and washed with boiling water until the washing was no longer acid to litmus. The entire residue was transferred back to the original digestion flask with a spatula, and the residue on the linen was washed into the digestion flask with 200 ml of boiled dilute 1.25% NaOH. The digestion flask was again boiled for 30 min with constant shaking, and the liquid level was maintained as before. At the end of 30 min, the residue was washed thoroughly with boiling water till free from alkali and was transferred to a prepared Gooch crucible and dried to a constant weight at 105°C in a GZX‐9240 MBE hot‐air oven (Shanghai Boxun Industrial Co., Ltd., China). The weight of the empty Gooch crucible was recorded (*W* [g]) before use. The residue was again washed with 15 ml of 95% ethyl alcohol. The content in the Gooch crucible was dried at 105°C, cooled in a desiccator, and weighed to a constant weight (*W*_1_ [g]). The Gooch crucible and its contents were transferred to a muffle furnace and ignited at 600 ± 20°C for 1 hr until all carbonaceous matter was burnt. The Gooch crucible containing the ash was cooled in a desiccator and weighed (*W*_2_ [g]). The percentage of crude fiber of the HS sample was calculated with the following formula:

(8) $$\%\text{Crude}\;\text{fiber}=\frac{\left(W_{1}‐W\right)‐\left(W_{2}‐W\right)}{\text{weight}\;\text{of}\;\text{sample}}\times 100,$$

where *W* is the weight of the empty Gooch crucible, *W*_1_ is the weight of the Gooch crucible and oven‐dried sample after washing with 95% ethyl alcohol, and *W*_2_ is the weight of the Gooch crucible and ash.

The ash content was determined by weighing 5 g of an HS flour sample into a clean, dried, and previously weighed empty crucible with a lid (*W*_1_ [g]). The weight of the sample and the empty crucible with the lid was recorded (*W*_2_ [g]). The sample was ignited over a low flame to char (carbonize) the organic matter with the lid removed. The crucible was then placed in a muffle furnace at 600°C for 6 hr until it ashed completely. It was then transferred directly to the desiccator, cooled, and weighed with the lid immediately (*W*_3_ [g]). The ash content of the HS sample was calculated with the following formula:

(9) $$\%\text{Ash}\;\text{content}=\frac{W_{3}‐W_{1}}{W_{2}‐W_{1}}\times 100,$$

where *W*_1_ is the weight of the empty crucible with the lid, *W*_2_ is the weight of the crucible with the lid and sample, and *W*_3_ is the weight of the crucible with the lid and ash.

### 2.7 TPC, TFC, TPAs, and antioxidant activity determination

The total phenolic content was determined using the Folin–Ciocalteu method as described by Chaves et al. (2020) with slight modification. Five milliliters of freshly prepared Folin–Ciocalteu reagent (1:1 v/v) was added to 500 μl of HS extract. Afterward, 5 ml of 7.5% Na_2_CO_3_ (w/v; 75 g/L) was added, vortexed for 10 s, and incubated at 25°C for 30 min. The absorbance was measured at 760 nm using a UV‐1600 spectrophotometer (Beijing Rayleigh Analytical Instrument, China) against a blank (distilled water and reagents). A standard curve was plotted using gallic acid (15.125–500 µg/ml) as a standard. The total phenolic content was expressed as milligrams of gallic acid equivalent per gram of HS sample.

Total flavonoid content was determined by the aluminum chloride colorimetric assay as described by Mitrevska et al. (2020) with modifications. Three hundred microliters of 5% NaNO_2_ (w/v; 50 g/L) and 4 ml of distilled water were added to 1 ml of HS extract in succession, vortexed for 15 s, and allowed to stand for 5 min. Afterward, 300 μl of 10% AlCl_3_ (w/v; 100 g/L) was added, vortexed, and allowed to stand for another 1 min. Two milliliters of 1 M NaOH was then added, and the volume was adjusted to 10 ml with 2.4 ml of distilled water. The mixture was incubated at 25°C for 10 min with intermittent shaking. The absorbance was measured at 510 nm using a UV‐1600 spectrophotometer (Beijing Rayleigh Analytical Instrument, China). A standard curve was plotted using rutin (1.5625–50 µg/ml) as a standard. The results were expressed as milligrams of rutin equivalent per gram of HS sample.

The antioxidative activity of the HS variety was determined by 2,2‐diphenyl‐1‐picrylhydrazyl (DPPH) radical scavenging activity and ferric reducing antioxidant power (FRAP) assays.

The DPPH radical scavenging activity of HS was determined according to the protocol outlined by Chaves et al. (2020) with slight modifications. One milliliter of HS extract was added to 2 ml of a 0.1 mM DPPH solution (prepared using methanol). After vortexing, the mixture was incubated in the dark for 30 min at 25°C. The absorbance was measured at 517 nm using a UV‐1600 spectrophotometer (Beijing Rayleigh Analytical Instrument, China). The linear range for the ascorbic acid standard used was 12.5–800.0 μg/ml. The result was expressed as μmol AA eq/g dry HS sample.

The FRAP assay was conducted according to the procedure reported by Chaves et al. (2020) with slight modifications: 3.8 ml of the FRAP reagent (a mixture of 10 parts 300 mM sodium acetate buffer [pH 3.6], 1 part 10 mM 2,4,6‐tripyridyl‐s‐triazine [TPTZ] in 40 mM HCl, and 1 part 20 mM FeCl_3_·6H_2_O) was added to 200 μl of HS extract. The resulting solution was incubated for 30 min at 37°C. The absorbance was measured at 593 nm using a UV‐1600 spectrophotometer (Beijing Rayleigh Analytical Instrument, China). The results were expressed in milligram equivalents of FeSO_4_ per dry weight using a FeSO_4_ standard curve generated under the same conditions. The calibration line was established using the following concentrations of FeSO_4_: 0.0025, 0.005, 0.0075, 0.01, and 0.02 mg/ml.

Total phenolic acids were determined with the method described by Haida and Hakiman (2019) with slight modifications. Briefly, 1 ml of HS extract was added to 9 ml of distilled water in a test tube. Then, 1 ml of Folin–Ciocalteu phenol reagent was added to it, and the mixture was mixed thoroughly with a vortex. After 5 min, 10 ml of 7% sodium carbonate was added. Next, 4 ml of distilled water was added, and the mixture was adjusted to a final volume of 25 ml. The reaction mixture was incubated for 90 min at room temperature, and the absorbance was measured at 750 nm using a UV‐1600 spectrophotometer (Beijing Rayleigh Analytical Instrument, China). The TPAs were expressed as milligrams of gallic acid equivalents per gram of HS. A standard curve for gallic acid (as standard) in methanol was prepared using different concentrations (100–700 μg/ml).

### 2.8 CA determination

Chlorogenic acid determination was performed according to the method of Adane et al. (2019). Forty milligrams of an HS sample was weighed and dissolved in 30 ml of distilled water in a 100‐ml beaker. The solution was stirred for 30 min using a magnetic stirrer (model C‐MAG HS 7 S025, IKA, Germany) and heated (at 40°C) to increase the solubility of CA in the solution. The solution was filtered through double‐loop qualitative filter paper (No. 1568, Ge Biotechnology Co. Ltd., China) to remove particles from the solution. The filtrate containing CA was collected and measured to obtain the volume of the sample extract. The absorbance of the measured sample extract was taken using a UV‐1601 spectrophotometer (Beijing Rayleigh Analytical Instrument Co., Ltd., China) within the wavelength range of 190–1100 nm. The CA concentration was computed against the standard solution using Beer–Lambert's Law at λ_max_ = 325 nm (the maximum wavelength). The CA content and the percentage of CA of the HS sample were calculated using Equations (10) and (11) (Adane et al., 2019), respectively:

(10) $$\text{CA}\;\text{content}\;\left(\text{mg}\right)=\frac{\left[\text{CA}\;\text{conc}\;\left(\text{mg}/L\right)\right]\times {\left[\text{total}\;\text{sample}\;\text{volume}\;\left(\text{ml}\right)\right]}^{2}}{\left[\text{measured}\;\text{sample}\;\text{volume}\;\left(\text{ml}\right)\right]\times 1000},$$

(11) $$\%\text{CA}\;\left(\frac{w}{w}\text{\%}\;\right)=\frac{\left[\text{calculated mass of CA}\;\left(\text{mg}\right)\right]}{\left[\text{mass of sample measured}\;\left(\text{mg}\right)\right]}\times 100\%,$$

### 2.9 Statistical analysis

Design Expert software (version 11.0.5.0, STAT‐EASE, Inc., USA) was used for both the experimental designs and the statistical analysis. A *P*‐test, the coefficient of determination (*R* ^2^), the coefficient of variation (CV), and a lack‐of‐fit test at *p* < .05, .01, and .001 were used to assess the accuracy of the model. Data were reported as the mean ± standard deviation of three independent determinations. MINITAB software (version 18.1; Minitab, Inc., USA) was used to compute an analysis of variance with Tukey's test applied at *p* < .05 to compare the means. Correlations between parameters were computed with Pearson's correlation. Relationships between parameters were computed with principal component analysis (PCA) using OriginPro 2018 software (OriginLab, Inc., USA).

## 3 RESULTS AND DISCUSSION

Ultrasonic‐assisted extraction was performed to investigate the extraction of bioactive CA with a high yield from the HS variety. Four ultrasonic factors were experimented with at three levels using the BBD to obtain a high yield of CA with enhanced DPPH radical scavenging activity from the HS sample (Table 1). A quadratic polynomial model was fitted to the dependent parameters. Analysis of variance was applied to evaluate the effects and interactions of the factors and the model significance statistically. Table 2 shows the model *p* and *F* values of the regression coefficients of the response parameters. Generally, factors exhibiting high significant effects have greater *F* values and smaller *p* values (Zhou et al., 2015). The large *F* values indicated a significant model, and there was only a 0.01% chance that it could occur by noise. Similarly, the high *R* ^2^ values of 0.9967 (for CA yield) and 0.9239 (for DPPH radical scavenging activity) indicated a significant model. It also indicated that 92.39% and 99.67% of the total variations for the DPPH radical scavenging activity and CA yield, respectively, were due to the independent parameters. Furthermore, the lack‐of‐fit values of the models were insignificant statistically. The quadratic polynomial model, therefore, was a good estimation (model fitness) of the responses and explained the responses adequately.

**TABLE 1:** Box–Behnken design matrix with the experimental design and data for the ultrasonic‐assisted extraction of CA from the heilong48 soybean variety

Run	Ultrasonic parameters (actual and coded values)				CA yield (mg/g)		DPPH (μmol AA eq/g dry sample)	
	Frequency (kHz)	Power density (W/L)	Temperature (°C)	Time (min)	Experimental values	Predicted values	Experimental values	Predicted values
	A	B	C	D	CA_(Actual)_	CA_(Predicted)_	DPPH_(Actual)_	DPPH_(Predicted)_
1	60 (+1)	40 (0)	40 (0)	35 (+1)	4.09 ± 0.01	4.03	90.93 ± 0.07	91.10
2	40 (0)	40 (0)	40 (0)	30 (0)	2.52 ± 0.01	2.44	92.92 ± 0.09	92.82
3	40 (0)	40 (0)	45 (+1)	25 (−1)	3.06 ± 0.02	3.07	91.11 ± 0.03	91.17
4	40 (0)	30 (−1)	35 (−1)	30 (0)	3.97 ± 0.01	3.92	91.50 ± 0.16	91.52
5	40 (0)	50 (+1)	40 (0)	35 (+1)	4.79 ± 0.02	4.81	91.07 ± 0.03	91.26
6	60 (+1)	40 (0)	40 (0)	25 (−1)	4.01 ± 0.02	4.02	91.79 ± 0.21	92.18
7	20 (−1)	40 (0)	40 (0)	35 (+1)	3.15 ± 0.01	3.01	93.76 ± 0.03	93.42
8	60 (+1)	50 (+1)	40 (0)	30 (0)	4.43 ± 0.01	4.44	91.66 ± 0.06	91.63
9	60 (+1)	40 (0)	45 (+1)	30 (0)	4.85 ± 0.01	4.89	92.13 ± 0.09	92.07
10	40 (0)	50 (+1)	35 (−1)	30 (0)	3.52 ± 0.00	3.48	91.00 ± 0.03	91.02
11	40 (0)	40 (0)	35 (−1)	35 (+1)	3.19 ± 0.01	3.22	90.01 ± 0.12	90.02
12	20 (−1)	40 (0)	45 (+1)	30 (0)	2.49 ± 0.01	2.55	92.36 ± 0.06	92.60
13	40 (0)	40 (0)	40 (0)	30 (0)	2.42 ± 0.02	2.44	92.05 ± 0.03	92.82
14	20 (−1)	40 (0)	35 (−1)	30 (0)	4.44 ± 0.02	4.50	92.41 ± 0.12	92.36
15	40 (0)	40 (0)	35 (−1)	25 (−1)	2.99 ± 0.03	2.97	91.38 ± 0.10	91.75
16	60 (+1)	30 (−1)	40 (0)	30 (0)	4.86 ± 0.02	4.83	92.54 ± 0.09	92.43
17	40 (0)	40 (0)	45 (+1)	35 (+1)	2.30 ± 0.01	2.35	93.00 ± 0.06	92.69
18	20 (−1)	50 (+1)	40 (0)	30 (0)	3.97 ± 0.01	4.04	92.76 ± 0.09	92.94
19	40 (0)	30 (−1)	40 (0)	35 (+1)	1.34 ± 0.00	1.43	92.95 ± 0.09	93.22
20	20 (−1)	40 (0)	40 (0)	25 (−1)	3.56 ± 0.02	3.49	92.66 ± 0.09	92.54
21	40 (0)	30 (−1)	40 (0)	25 (−1)	4.99 ± 0.02	5.06	92.51 ± 0.07	92.20
22	20 (−1)	30 (−1)	40 (0)	30 (0)	3.66 ± 0.01	3.68	93.70 ± 0.03	93.80
23	40 (0)	40 (0)	40 (0)	30 (0)	2.39 ± 0.01	2.44	93.09 ± 0.12	92.82
24	60 (+1)	40 (0)	35 (−1)	30 (0)	3.68 ± 0.02	3.71	90.57 ± 0.10	90.21
25	40 (0)	40 (0)	40 (0)	30 (0)	2.48 ± 0.02	2.44	92.99 ± 0.14	92.82
26	40 (0)	40 (0)	40 (0)	30 (0)	2.40 ± 0.01	2.44	93.03 ± 0.06	92.82
27	40 (0)	50 (+1)	45 (+1)	30 (0)	3.59 ± 0.02	3.52	91.70 ± 0.12	91.74
28	40 (0)	30 (−1)	45 (+1)	30 (0)	3.20 ± 0.03	3.11	92.86 ± 0.07	92.90
29	40 (0)	50 (+1)	40 (0)	25 (−1)	1.64 ± 0.02	1.65	92.89 ± 0.06	92.50

Abbreviations: CA, chlorogenic acid; DPPH, 2,2‐diphenyl‐1‐picrylhydrazyl.

**TABLE 2:** Analysis of variance, regression analysis, and optimum conditions for the ultrasonic‐assisted extraction of CA from the heilong48 soybean variety

Source	CA yield (mg/g)		DPPH (μmol AA eq/g dry sample)	
	*F* value	*p* value	*F* value	*p* value
Model	303.73	<.0001***	12.13	<.0001***
Linear				
A: Frequency	285.19	<.0001***	39.38	<.0001***
B: Power density	0.0844	.7757^NS^	15.15	.0016*
C: Temperature	69.77	<.0001***	24.16	.0002**
D: Time	25.48	.0002**	0.2347	.6355^NS^
Interactions				
AB	21.67	.0004**	0.0066	.9364^NS^
AC	385.17	<.0001***	4.75	.0469*
AD	9.50	.0081*	7.04	.0189*
BC	27.92	.0001***	0.7980	.3868^NS^
BD	1829.63	<.0001***	9.36	.0085*
CD	36.47	<.0001***	19.47	.0006**
Quadratic				
A^2^	1251.28	<.0001***	0.1253	.7287^NS^
B^2^	503.41	<.0001***	0.2171	.6484^NS^
C^2^	136.96	<.0001***	43.47	<.0001***
D^2^	9.07	.0093*	9.74	.0075*
Fitting statistics				
Lack of fit	2.44	.2028^NS^	0.6206	.7539^NS^
*R* ^2^	0.9967		0.9239	
Adjusted *R* ^2^	0.9934		0.8477	
Predicted *R* ^2^	0.9830		0.6867	
Adequate Precision	63.5276		14.2280	
C.V. %	2.35		0.4007	
Mean	3.38		92.18	
Standard Deviation	0.0795		0.3694	
Optimization equations				
$\begin{array}{cc} \text{CA}\;\text{yield}\left(\text{mg}/g\right) & =2.44+0.3875A‐0.1917C‐0.1158D\\ & \;+0.185\text{AB}+0.78\text{AC}+0.1225\text{AD}+0.21\text{BC}+1.7\text{BD}\\ & \;‐0.24\text{CD}+1.1A^{2}+0.7003B^{2}+0.3652C^{2}+0.094D^{2} \end{array}$				
$\begin{array}{cc} \text{DPPH}\left(\mu \text{mol}\;\text{AA}\;\text{eq}/g\;\text{dry}\;\text{sample}\right) & =92.82‐0.6692A‐0.415B+0.5242C\\ & \;+0.4025\text{AC}‐0.49\text{AD}‐0.565\text{BD}\\ & \;+0.815\text{CD}‐0.9563C^{2}‐0.4526D^{2} \end{array}$				

Note

*, **, and *** denote significance at *p* < .05, *p* < .01, and *p* < .001, respectively, whereas NS denotes not significant.

Abbreviations: CA, chlorogenic acid; DPPH, 2,2‐diphenyl‐1‐picrylhydrazyl.

### 3.1 Effect of ultrasonic parameters on the CA yield of ultrasound‐treated heilong48 soybean (UTHS) extract

The predictive equation obtained from the results for explaining the effectiveness of the ultrasonication pretreatments of HS extracts for optimal extraction of CA with high yields was written in coded terms after the removal of insignificant variables as follows:

(12) $$\begin{array}{cc} \text{Chlorogenic}\;\text{acid}\;\text{yield}\left(\text{mg}/g\right) & =2.44+0.3875A‐0.1917C‐0.1158D‐0.185\text{AB}\\ & \;+0.78\text{AC}+0.1225\text{AD}+0.21\text{BC}+1.7\text{BD}‐0.24\text{CD}\\ & \;+1.1A^{2}+0.7003B^{2}+0.3652C^{2}+0.094D^{2}. \end{array}$$

Among the linear terms, frequency (A) and temperature (C) were extremely significant factors that influenced CA yield and were followed by time (D), whereas the effect of power density (B) was insignificant though positive (Table 2). All the interaction and quadratic terms showed a very significant effect for CA yield (*p* < .01). The results showed that the experimental CA yield obtained varied from 1.34 ± 0.00 to 4.09 ± 0.01 mg/g; the maximum was achieved at A = 60 kHz, B = 40 W/L, C = 40°C, and D = 35 min, whereas the minimum was obtained at A = 40 kHz, B = 30 W/L, C = 40°C, and D = 35 min. This suggests that lower frequency and power density pretreatment (sonication) conditions may not be desirable for the ultrasonic‐assisted extraction of CA with high yields from the HS variety.

**FIGURE 1:**
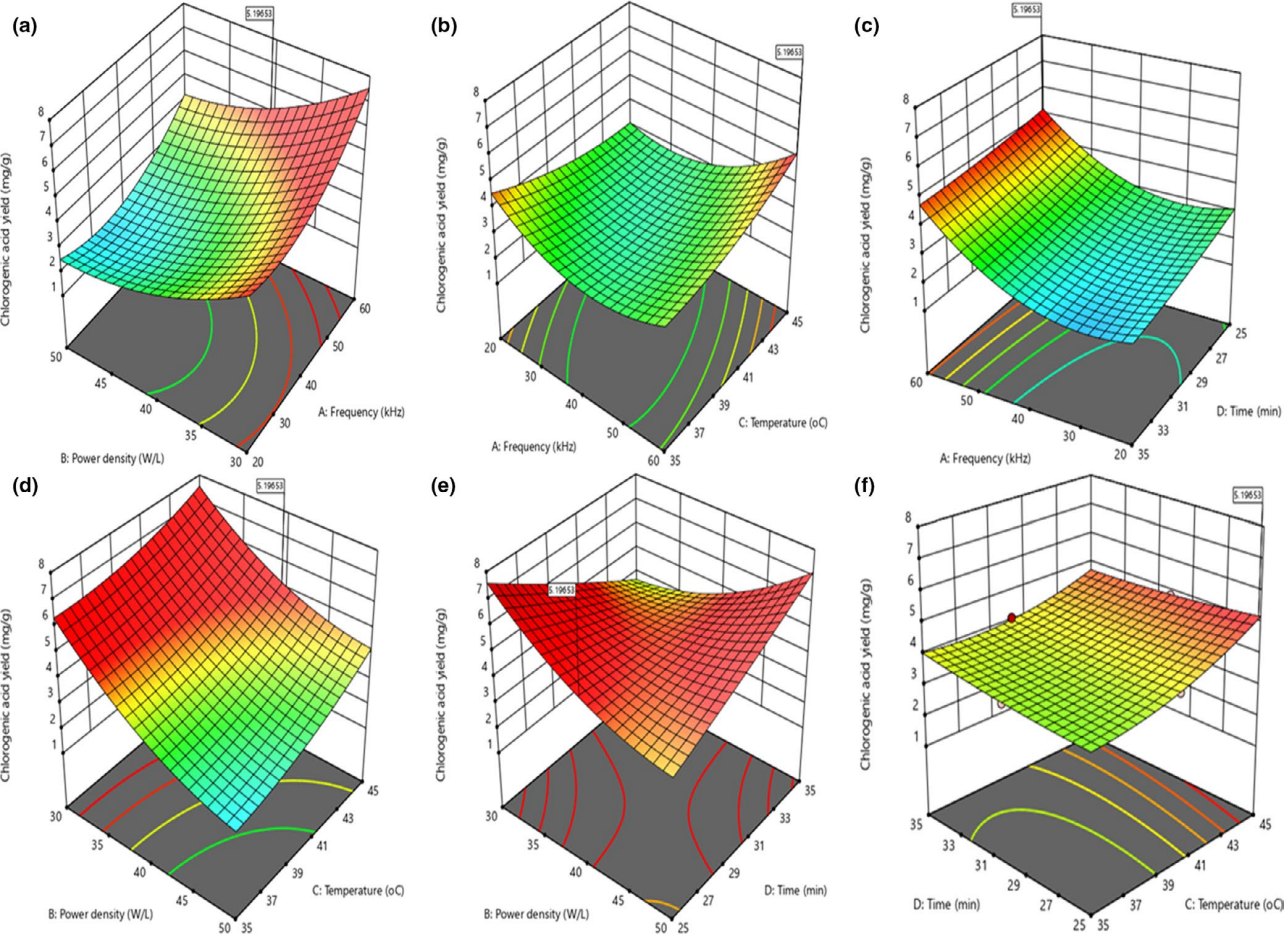
Contour and response surface plots of the interactive influence of frequency, power density, temperature, and time on the chlorogenic acid yield of the ultrasonic‐treated heilong48 soybean sample

Figure 1 displays three‐dimensional response surface (3‐D RS) and two‐dimensional contour (2‐D C) plots of the CA yield for the UTHS sample as a function of the factors (A, B, C, and D). Graphical representations of the regression equation (3‐D RS and 2‐D C plots) are very useful for evaluating independent–dependent (factor–response) variable relationships. A circular 2‐D C plot indicates insignificant interactions of parameters, whereas an elliptical 2‐D C plot depicts significant interactions of variables (Cui et al., 2014). The CA yield slightly decreased at first and then increased steadily as A increased and B decreased. The maximum CA yield was obtained when A and B were 60 kHz and 30 W/L, respectively. Similarly, the CA yield decreased slightly at first and then increased when both A and C increased; this is confirmed by the perturbation plot in Figure S1a. Figure 1 e shows that BD interactions were significant because of the elliptical 2‐D C plots, and this was confirmed by the results in Table 2. The CA yield increased with increasing C and D. This finding agrees with the report of Banožić et al. (2019).

### 3.2 Effect of ultrasonic parameters on the DPPH radical scavenging activity of the UTHS extract

The DPPH radical scavenging activity model obtained through a multiple regression analysis of the experimental data was reported in coded values after the elimination of insignificant terms by the quadratic polynomial equation as follows:

(13) $$\begin{array}{cc} \text{DPPH}\left(\mu \text{mol}\;\text{AA}\;\text{eq}/g\;\text{dry}\;\text{sample}\right) & =92.82‐0.6692A‐0.415B+0.5242C\\ & \;+0.4025\text{AC}‐0.49\text{AD}‐0.565\text{BD}\\ & \;+0.815\text{CD}‐0.9563C^{2}‐0.4526D^{2}. \end{array}$$

**FIGURE 2:**
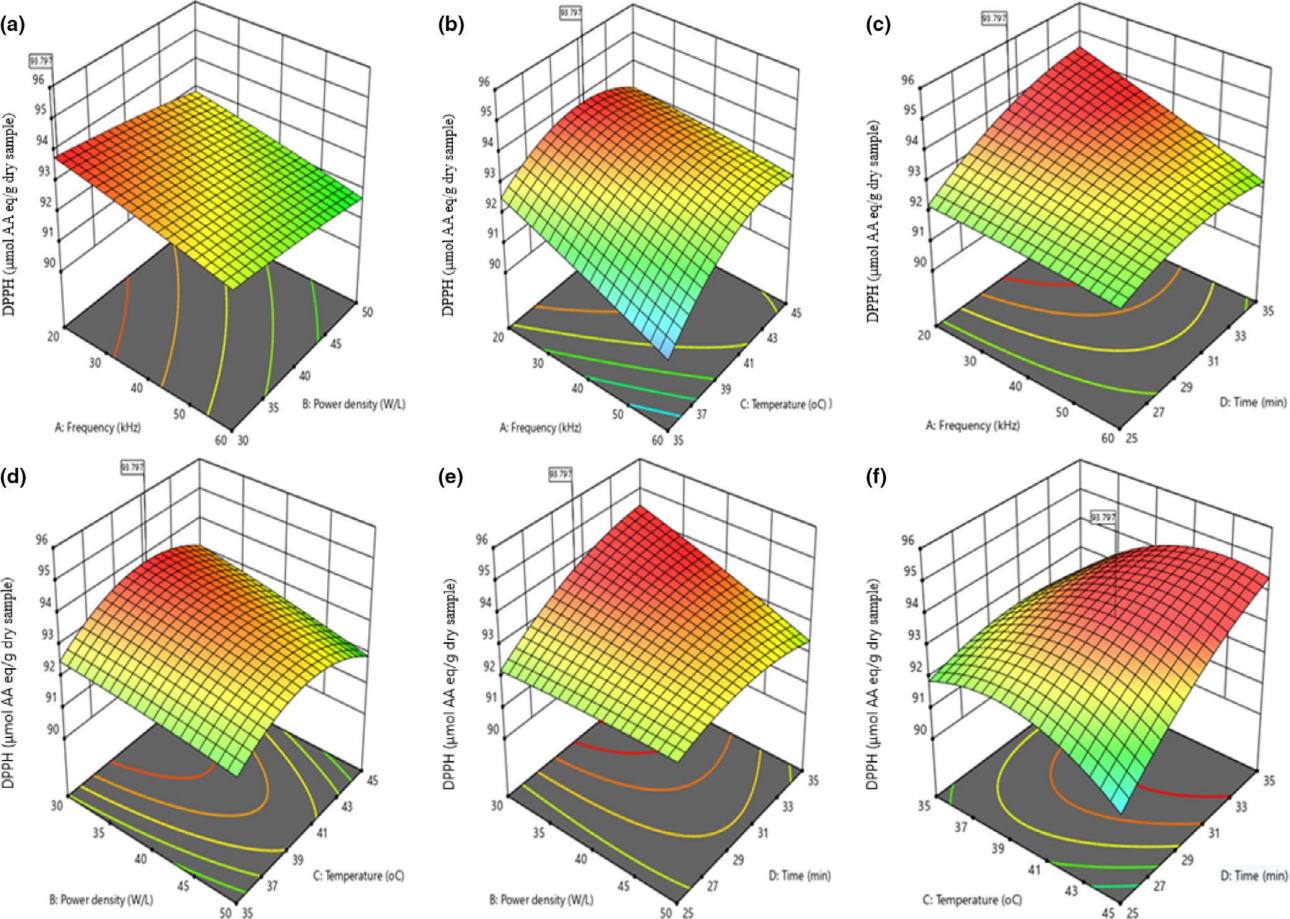
Contour and response surface plots of the interactive influence of frequency, power density, temperature, and time on the 2,2‐diphenyl‐1‐picrylhydrazyl (DPPH) of the ultrasonic‐treated heilong48 soybean sample

The linear terms A, B, and C significantly (*p* < .01) influenced the DPPH radical scavenging activity of the UTHS sample within a 99% confidence interval (Table 2). However, A was the extremely significant variable that influenced the DPPH radical scavenging activity of the UTHS sample. The results showed that the interactions between A and C (AC), A and D (AD), B and D (BD), and C and D (CD) significantly influenced the DPPH radical scavenging activity of the UTHS sample. This is confirmed by the graphs in Figure 2 with their elliptical 2‐D C plots. Consequently, as the frequency decreased and the temperature or time increased, the DPPH radical scavenging activity of the UTHS sample also increased (Figure 2b,c). Statistically, the quadratic terms of A and B did not influence the DPPH radical scavenging activity of the UTHS sample. Figure 2a–f shows that all six 3‐D RS plots were convex in shape. This suggests that the ranges of the parameters were correctly selected. Figure 2a,b,d,e shows that the DPPH radical scavenging activity of the UTHS sample steeply decreased when A and/or B increased, and this is confirmed by the perturbation plot in Figure S1b. The results showed that as the temperature increased, the DPPH radical scavenging activity of the UTHS sample also increased gradually, first to somewhere above midrange temperatures (41–43°C), and then it decreased from this point to 45°C (Figure 2d,f). From Figure 2c,e,f, the DPPH radical scavenging activity increased slowly with increasing D to a maximum at 35 min. A similar result was reported by Mintah et al. (2019).

### 3.3 Model validation and verification

The reliability of the model equations obtained for the prediction of the optimum response values (CA yield and DPPH radical scavenging activity: 4.990 ± 0.079 mg/g and 93.171 ± 0.369 μmol of AA eq/g dry sample, respectively) was tested under the obtained optimum conditions: A = 20.000 kHz, B = 30.000 W/L, C = 37.935°C, and D = 27.978 min. The optimum conditions were, however, modified to take into consideration the actual extraction operation as A = 20.0 kHz, B = 30.0 W/L, C = 37.9°C, and D = 28.0 min, under which the experimental DPPH radical scavenging activity and CA yield performed in triplicate were 93.197 ± 0.213 μmol of AA eq/g dry sample and 5.007 ± 0.033 mg/g, respectively. The relative error of the predicted and experimental values of the CA yield was 0.0034 (0.34%), and that of DPPH radical scavenging activity was 0.0002 (0.02%; Table S1b); these compared very well with the predicted values and were less than 5% (Boateng et al., 2020). This showed that the model was feasible and reliable and hence is acceptable for high bioactive CA extraction from the HS variety.

### 3.4 Comparison of CA yield and DPPH radical scavenging activity from UAE and extraction without ultrasonic treatment

**FIGURE 3:**
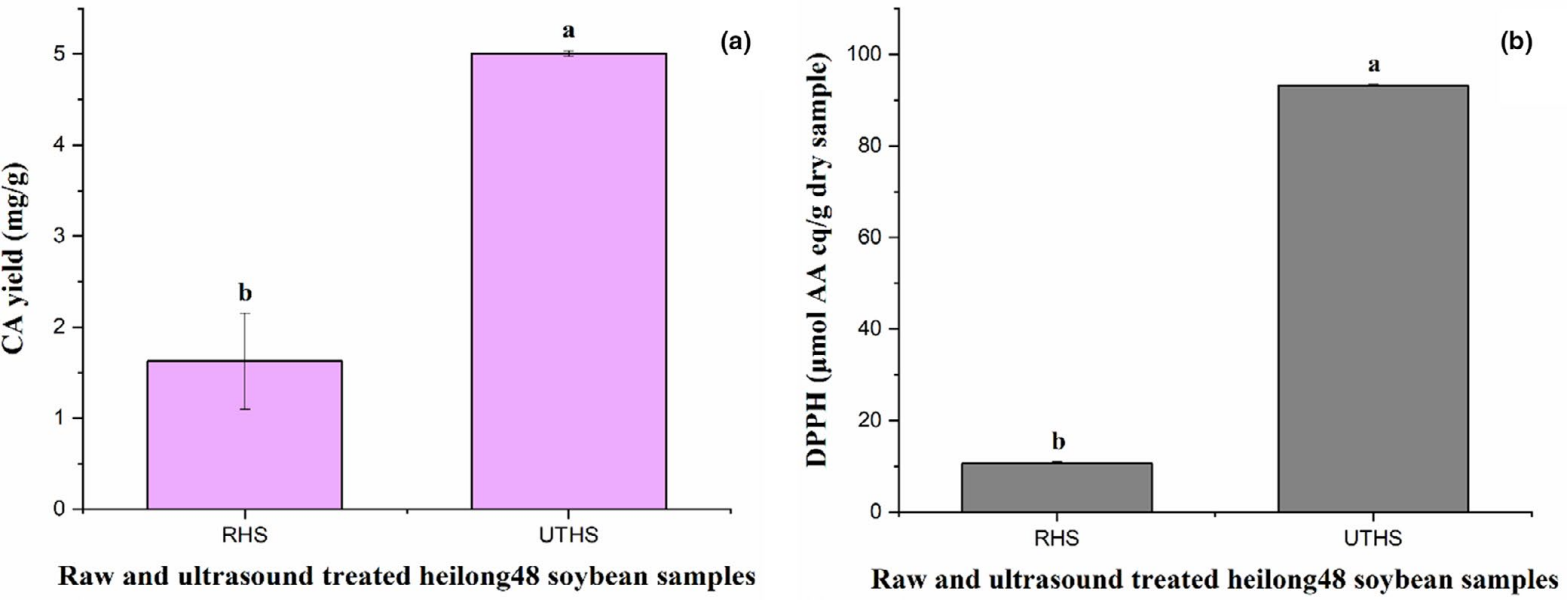
Chlorogenic acid yield (a) and DPPH (b) of raw heilong48 soybean (RHS) and ultrasound‐treated heilong48 soybean (UTHS) samples

The DPPH radical scavenging activity (93.197 ± 0.213 μmol of AA eq/g dry sample) and the CA yield (5.007 ± 0.033 mg/g) obtained under the optimized UAE conditions (frequency = 20.0 kHz, power density = 30.0 W/L, temperature = 37.9°C, and time = 28.0 min) were compared with those obtained without ultrasonic treatment (DPPH radical scavenging activity, 10.760 ± 207 μmol of AA eq/g dry sample; CA yield, 1.627 ± 0.528 mg/g) as depicted in Figure 3. The results indicated that UAE produced a higher CA yield with improved DPPH radical scavenging activity in comparison with extraction without ultrasounic treatment. This was in accordance with the report of Mintah et al. (2019). Ultrasonication involves back and forth oscillation and collapsing of cavitation bubbles, which lead to shear thrusts, microjets, shock waves, and turbulence. The intense circulatory motion of the cavitation bubbles leads to microstreaming, which causes cell wall disruption (for food samples), food matrix permeability, and mass transfer (Mintah et al., 2019). This implies that the cellular matrix of the UTHS sample was more permeable after the ultrasonic treatment and allowed the extraction solvent into its internal structure, which caused more CA to dissolve in solution; this facilitated the extraction of CA with a high yield and enhanced antioxidative activity from the UTHS sample.

### 3.5 Effect of UAE on the structure of the UTHS sample

**FIGURE 4:**
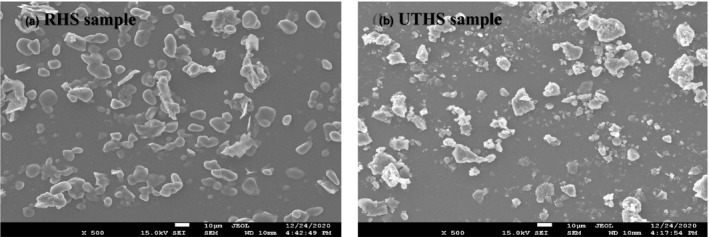
Scanning electron microscopy micrograph of raw heilong48 soybean (RHS) and ultrasound‐treated heilong48 soybean (UTHS) samples

Figure 4 depicts the microstructure of the HS samples: non–ultrasound‐treated (raw heilong48 soybean [RHS]) and ultrasound‐treated under the obtained optimized conditions (UTHS). The particles of the RHS sample were characteristically compact and smoothly surfaced. The particles of the UTHS sample were, however, roughly surfaced, porous, and loose. This indicates that the cavitation influence of the optimized ultrasonic conditions caused a sponge influence, and this resulted in the loose structure of the UTHS sample. The cavitation also broke the bonds (covalent, noncovalent, hydrogen, and disulfide) between the molecules of the cell wall of the UTHS sample, which increased free CA mass flow. This explains the higher CA yield with the improved antioxidative activity obtained from the UTHS sample. These results are in agreement with those reported by Mintah et al. (2019).

### 3.6 Proximate composition of HS

The proximate composition of the HS variety was 42.583 ± 1.010% crude protein, 18.733 ± 0.116% crude fat, 4.587 ± 0.012% ash, 1.815 ± 0.005% crude fiber, and 7.650 ± 0.150% moisture (Table 3). These components give interconnected information about food quality (Desseva et al., 2020). The fat content was less than that reported by Rehal et al. (2019), Muhsin and Zegeye (2018), and (Etiosa et al., 2018) but was in agreement with that reported by Dixit et al. (2011). Likewise, the protein content agreed with that reported by Rehal et al. (2019) and Grieshop and Fahey (2001) but was less than that reported by Muhsin and Zegeye (2018) and Etiosa et al. (2018). The HS variety showed high protein and fat content. Proteins play a major role in the growth and maintenance of the human body and are, along with lipids and carbohydrates, the energy‐giving nutrients in food (Twinomuhwezi et al., 2020). As a result, these high protein and fat contents of the HS variety obtained showed that it is a rich dietary protein (Dixit et al., 2011) and food energy source for human nutrition.

**TABLE 3:** Physicochemical properties of the heilong48 soybean variety

Parameter	Mean ± *SD* (%)
Moisture content	7.650 ± 0.150^d^
Total solids	92.351 ± 0.150^a^
Crude protein	42.583 ± 1.010^e^
Ash	4.587 ± 0.012^b^
Crude fat	18.733 ± 0.116^c^
Crude fiber	1.815 ± 0.005^f^

Note

Superscripts that do not share the same letter are significantly different at *p* < .05.

The moisture content and ash were comparable to those reported by Etiosa et al. (2018). However, they were higher than those reported by Muhsin and Zegeye (2018) and Rehal et al. (2019). The water/moisture content and/or total solids of food are significant not only for expressing on a dry or wet basis the content of the other constituents but also as a significant factor in the quality and stability of food (Twinomuhwezi et al., 2020). The low moisture content implied that under good storage conditions, the HS variety could have an extended shelf life. The HS variety had a high total solids content of 92.351 ± 0.150%. High total solids increase the nutritive value of products and improve the keeping quality (Odu et al., 2012). This suggests that the HS variety had a high nutritive value with improved keeping quality. The ash content suggests that the HS variety was a good source of valuable minerals. The crude fiber of the HS variety obtained was lower than the values of 6.732 ± 0.013% and 7.001 ± 0.668% for the Clark 63K and SCS 1 soybean varieties, respectively, reported by Muhsin and Zegeye (2018) and the value of 5.44% reported by Etiosa et al. (2018).

The proximate composition of the HS variety obtained showed that its quality was high. Nevertheless, food components are interrelated. Consequently, the proximate components of the HS variety are interrelated with its bioactive components. The most studied interaction is the protein–polyphenol interaction (Sęczyk et al., 2019). There is also a protein–CA interaction reported by Wildermuth et al. (2016) in which low‐molecular‐weight polypeptides are bonded to soluble CA (about half). This suggests that some of the CA in the HS bonded to its proteins; this thus resulted in a decreased quantity of free CA in the HS and would reduce the yield of CA extraction. This implies that when the protein content of HS increases, the yield of CA extraction will decrease.

### 3.7 Bioactive compounds of HS

The TPC of the HS variety was 4.726 ± 0.002 mg GAE/g (Table 4). These results were higher than the results of Malenčić et al. (2008) and Tepavčević et al. (2010) and agreed with the results reported by Lee et al. (2020) This implies that the HS variety had high TPC. However, the TFC of the HS variety was 0.040 ± 0.008 mg RE/g. This result was less than that reported by Prvulović et al. (2016). Nevertheless, Malenčić et al. (2012) reported a similar result. According to Maksimovíc et al. (2005), soybean hybrids are poor in flavonoids in comparison with some other industrial crops such as maize hybrids. This probably explained the low TFC obtained in this study. The TPC of the HS variety was significantly higher than the TFC. A similar trend was reported by Prvulović et al. (2016). According to Aberoumand and Deokule (2008), plant polyphenols through the hydrogen‐donating property of their hydroxyl groups act as reducing agents and antioxidants. This suggests that the HS variety is a good source of plant antioxidants.

**TABLE 4:** One‐way analysis of variance of the bioactive compounds and antioxidant activity of the heilong48 soybean variety

Parameter	Mean ± *SD*
Total flavonoid content (mg RE/g)	0.040 ± 0.008^f^
Total polyphenol content (mg GAE/g)	4.726 ± 0.002^c^
Total phenolic acids (mg GAE/g)	1.883 ± 0.005^d^
Chlorogenic acid content (mg/g)	1.627 ± 0.528^e^
DPPH (μmol AA eq/g dry sample)	10.760 ± 0.207^b^
FRAP (mg FeSO_4_/mg dry weight)	12.563 ± 0.280^a^

Note

Superscripts that do not share the same letter are significantly different at *p* < .05.

Abbreviations: DPPH, 2,2‐diphenyl‐1‐picrylhydrazyl; FRAP, ferric reducing antioxidant power.

Martins et al. (2011) stated that more than half of the 8000 phenolic compounds that occur naturally, flavonoids constitute the major component. Nevertheless, this study had opposing results to our finding/result and implied that the HS variety was richer in phenolic acids (with a CA content of 1.627 ± 0.528 mg/g) than flavonoids. The CA content of the HS variety obtained was lower than the value of 62.10 ± 0.13 mg/g reported by Adane et al. (2019) in the beans and leaves of *Coffea arabica*. The TPA of the HS variety was 1.883 ± 0.005 mg GAE/g (Table 4). Our result was higher than the values of 289.4 µg/g and 32 µg/g reported by Kumari and Chang (2016) and Perumalla and Nayeem (2012), respectively. This implies that the HS variety is a suitable raw material for the extraction of CA. The result, therefore, strongly suggests that the polyphenol content of the HS variety is high, and it should be considered as an important raw material for CA extraction, as its pharmacological effects (diuretic, anti‐inflammatory, or antioxidative activity) could be attributed to the presence of these valuable components.

### 3.8 Antioxidant activity of the HS variety

The antioxidative activity of soybean foods and seeds correlates with isoflavones and total phenolics positively (Devi et al., 2009). The antioxidant activity of the HS variety is shown in Table 4. This study used DPPH radical scavenging activity and FRAP in the analysis of the antioxidant activity of the HS variety; this was justified because one assay is inadequate to measure the antioxidant activity reported in literature (Xiao et al., 2014). Xiao et al. (2014) reported that a single antioxidant property model hardly reflects the antioxidant capacity of samples because of the differences in the theoretical bases of different antioxidant measurements. A single assay, therefore, could be inadequate. The antioxidant activity of the HS variety was 10.760 ± 0.207 μmol of AA eq/g dry sample (measured by the DPPH radical scavenging activity assay) and 12.563 ± 0.280 mg FeSO_4_/mg dry weight (measured by the FRAP assay). The DPPH radical scavenging activity result agrees with that reported by Prvulović et al. (2016), and the FRAP result was higher than that reported by Lee et al. (2020). Because soybeans contain different types of phenolic compounds, there is the possibility that their content and composition will influence the antioxidative activity level. There was a significant difference between DPPH radical scavenging activity and FRAP (*p* < .05). This trend was reported by Lee et al. (2020) and Prvulović et al. (2016). The high antioxidant activity exhibited by the HS variety suggests that it is a rich source of easily accessible plant‐based natural antioxidants that could be used as a potential food supplement or in the pharmaceutical industry.

### 3.9 Correlation between TPC, TPAs, CA, TFC, DPPH radical scavenging activity, and FRAP

**FIGURE 5:**
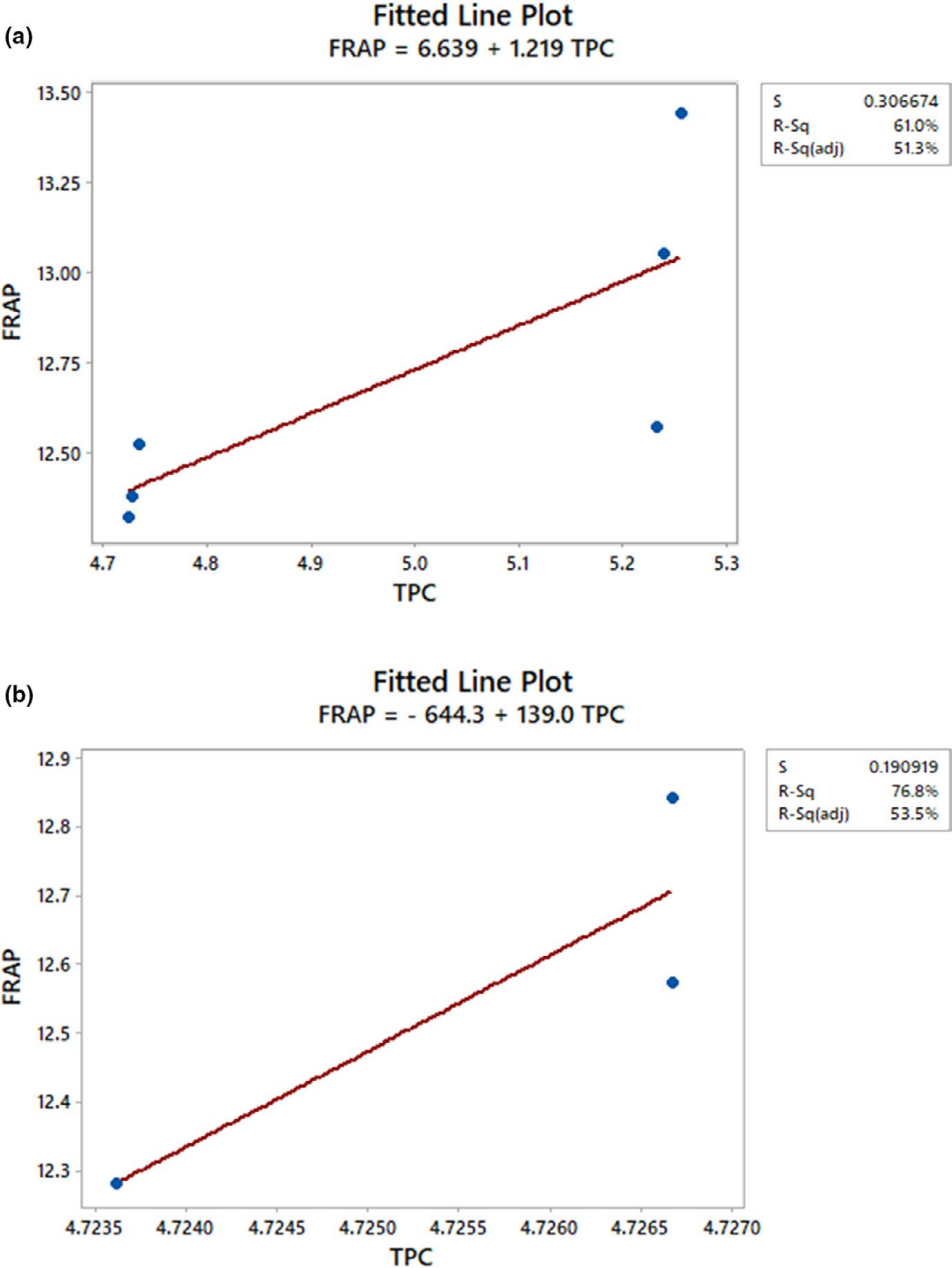
Correlation [(a) *r* ^2^ = 0.610; (b) *r* ^2^ = 0.768] between the total polyphenol content (TPC) and the ferric reducing antioxidant power (FRAP) of the heilong48 soybean variety

**FIGURE 6:**
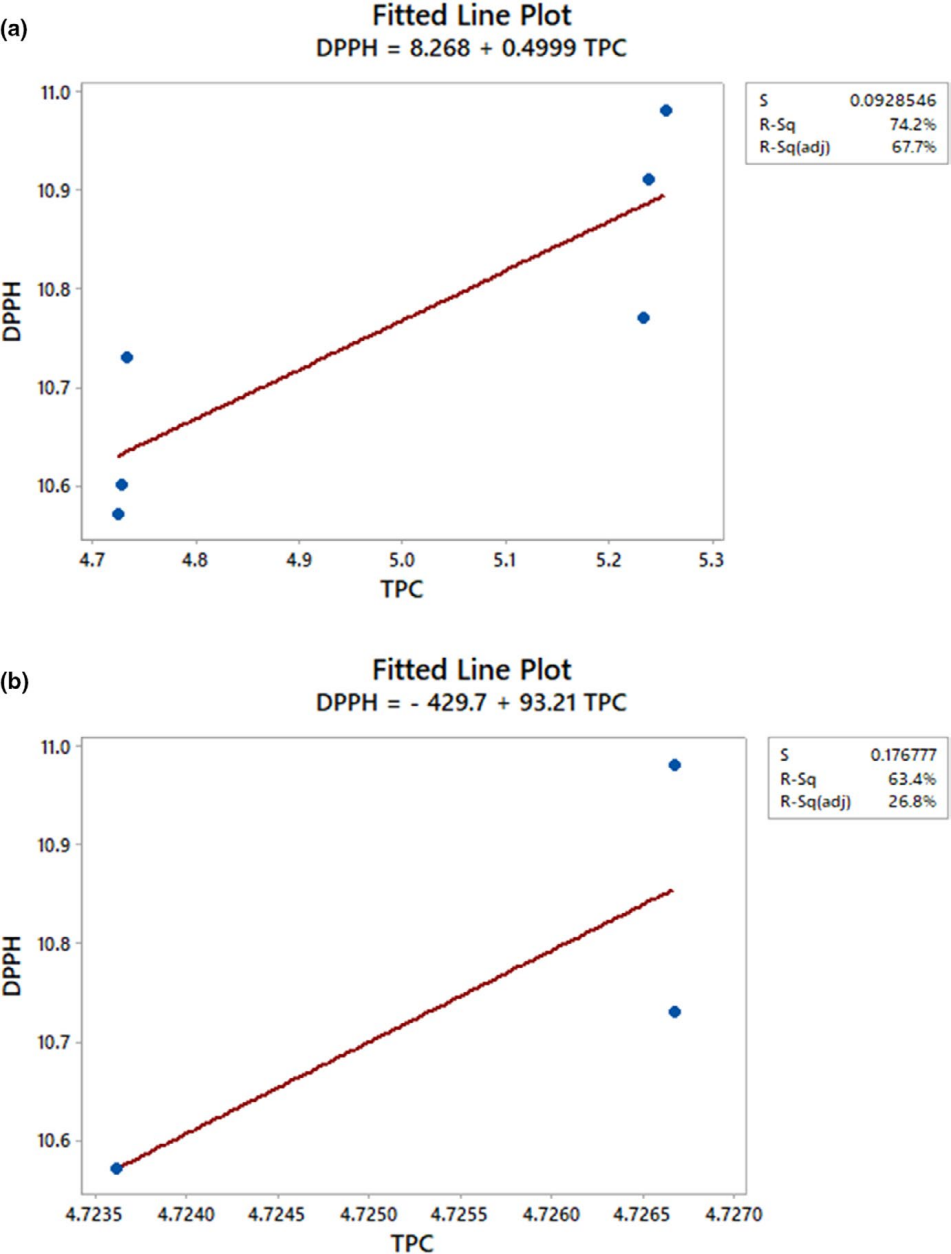
Correlation [(a) *r* ^2^ = 0.742, (b) *r* ^2^ = 0.634] between the total polyphenol content (TPC) and the 2,2‐diphenyl‐1‐picrylhydrazyl (DPPH) radical scavenging activity of the heilong48 soybean variety

The high TPC of the HS variety produced high antioxidative activity, which was shown by the strong correlation between TPC and antioxidative activity. Overall, the correlation between FRAP and TPC was stronger than that between DPPH free radical scavenging activity and TPC (Table S2a). The TPC was highly correlated with scavenging ability against DPPH radicals (*r* = 0.796) and FRAP (*r* = 0.876), and this was consistent with the report of Prvulović et al. (2016). Antioxidative activity increased proportionally with the TPC (Figures 5a,b and 6a,b). A linear correlation (*p* < .05) was established between DPPH radical scavenging activity and TPC with *r* ^2^ = 0.634 when three data points were used, but *r* ^2^ was 0.742 when six data points were plotted. This result agrees with that of Malenčić et al. (2008), who obtained *r* = 0.66963 (in seed extracts of different soybean hybrids) for multiple data points plotted. Similarly, FRAP had a linear relationship (*p* < .05) with TPC but with a higher *r* ^2^ value (*r* ^2^ = 0.768) in comparison with that of DPPH radical scavenging activity (Figures 5 and 6) when three data points were used. However, a lower *r* ^2^ value (*r* ^2^ = 0.610) in comparison with that of DPPH radical scavenging activity was obtained when six data points were plotted.

The high FRAP and DPPH radical scavenging ability of the HS variety obtained were due to the presence of high phenolic contents of the studied sample, and this was in agreement with the reports of Lee et al. (2020) and Xiao et al. (2014). This relationship between the antioxidative activity and TPC of the HS variety indicates that phenolic compounds contributed about 74% (by DPPH radical scavenging activity) or 61% (by FRAP) of the antioxidant activity of the HS variety. Generally, fewer than five data points are not enough to measure a strong correlation. Five or more data points are better and establish a stronger and significant correlation. This indicates that DPPH radical scavenging activity was a better parameter for measuring the antioxidant activity of the HS variety than FRAP. Hence, even though both DPPH radical scavenging activity and FRAP had a significant positive correlation with TPC, DPPH radical scavenging activity had a stronger relationship with TPC than FRAP.

Both FRAP and DPPH radical scavenging activity are measures of nonenzymatic antioxidative activity (Haida & Hakiman, 2019). As a result, the linear relationship obtained between FRAP and TPC showed that the HS variety had high antioxidant activity. According to Nam et al. (2014), the antioxidant activity of soybeans varies consistently with their genotype or ecological condition. Hence, the differences in our findings with respect to those of Nam et al. (2014)might be due to this reason. However, the strong correlations between the antioxidant activity and TPC of the HS variety support the overall opinion that phenolic compounds are the most significant antioxidants of plant materials. The DPPH radical scavenging activity assay is the most commonly used method for antioxidant activity measurement. This method differs in terms of kinetics and experimental conditions from FRAP. According to Ozgen et al. (2006), the DPPH radical scavenging activity method is more suited for samples with lipophilic antioxidants or those with a high lipid content. This might be the reason for the observed trend of correlations obtained between DPPH radical scavenging activity and TPC and between FRAP and TPC.

### 3.10 Protein–fat–polyphenol interaction

Phenolic compounds can have harmful nutritional influences by bonding with carbohydrates, lipids, and proteins (Alu’datt et al., 2013). Earlier studies by Bravo et al. (1992) reported frequent interactions between phenolic compounds and other components of plants (lipids, carbohydrates, and proteins) . Research shows that the chemical formation of phenolic compounds makes it possible for interactions to occur between them and other food components via hydrophobic interactions, ionic bonding, hydrogen bonding, and covalent bonding (Alu'datt et al., 2019). Equation (14) shows the interaction between the protein, fat, and TPC of the HS variety assessed by multiple regression (*r* ^2^ = 1.000). Figure S2a and Figure S2b show the individual correlations between protein and TPC and between fat and TPC, respectively:

(14) $$\text{TPC}=4.800000‐\left[0.001747\;\text{Protein}\;\text{content}\right]‐\left[0.000000\;\text{Fat}\;\text{content}\right],$$

The results show that both protein and fat negatively correlated with total polyphenol, and this implies that an increase in protein and fat content results in a decrease in TPC. Protein, however, had a complete or perfect correlation (*r* ^2^ = 1.000) with TPC (a strong relationship), whereas the correlation (*r* ^2^ = 0.246) between fat and TPC was weak (a weak relationship); this was consistent with the report of Akoglu (2018). This is also shown in the magnitude of their regression coefficients in the multiple regression equation (Equation 14). This result conforms with the findings of Alu’datt et al. (2013). Hence, there was an interaction between the protein and TPC of the HS variety, and this limited the quantity measured. This possibly indicates that the actual TPC of the HS variety was higher than that obtained and suggests the need to isolate protein from the bean before polyphenol or CA extraction.

### 3.11 Influence of protein and fat on the TPC and antioxidant activity of the HS variety

**FIGURE 7:**
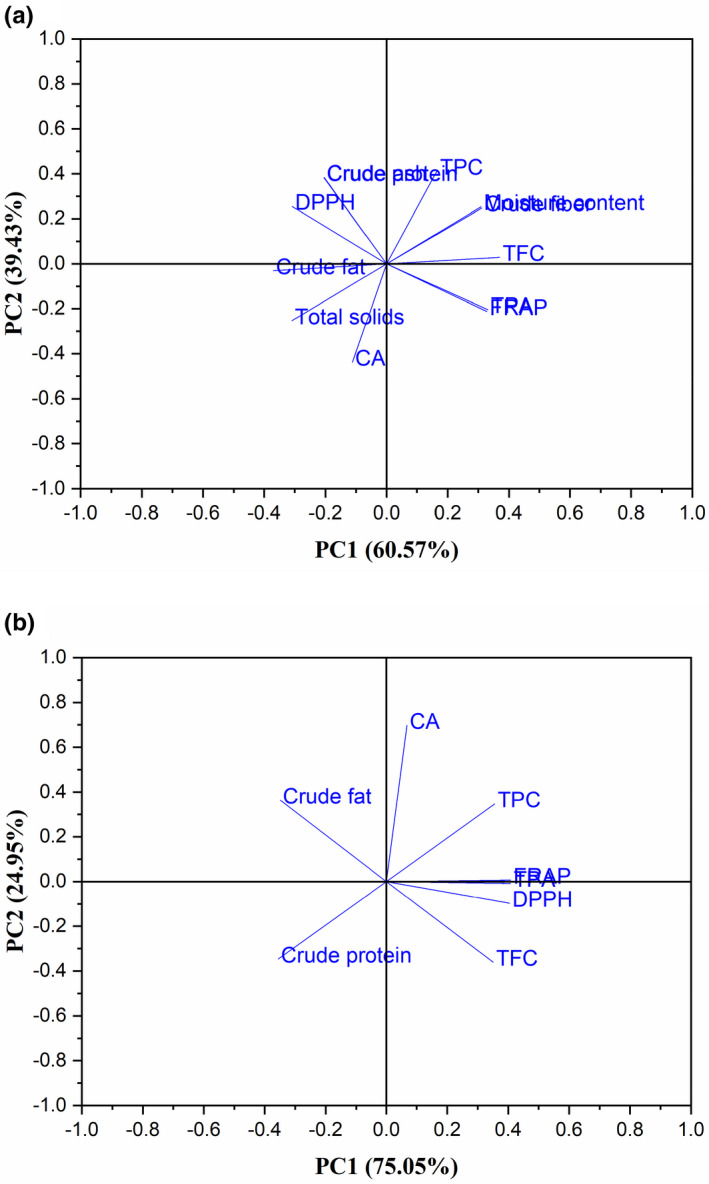
Principal component analysis: (a) loading plot of the moisture content, total solids, crude protein, ash, crude fat, crude fiber, TPC, TPA, CA, TFC, DPPH, and FRAP of the heilong48 soybean variety and (b) loading plot of the crude protein, crude fat, TPC, TPA, CA, TFC, DPPH, and FRAP of the heilong48 soybean variety

Principal component analysis displays similarities and differences among variables from their spatial distribution. Principal component analysis is statistically a useful technique. According to Kumari and Chang (2016) and Mishra et al. (2013), it effectively reduces the number of original variables (moisture content, total solids, fat, protein, ash, crude fiber, TPC, TPAs, CA, TFC, DPPH radical scavenging activity, and FRAP) with respect to principal components in one‐ or two‐dimensional plots and offers an insightful visual description of the group differences or clustering and outliers. Protein and fat values were considered as independent variables, whereas TPC, TPAs, CA, DPPH radical scavenging activity, and FRAP were the dependent variables. Parameters were centered on two main principal components: principal component one (PC1), which explained 60.570% of the variability among the data, and principal component two (PC2), which explained 39.430% of the variability among the data (Figure 7a). Thus, PC1 and PC2 explained 100.000% of the total data variance in the data set of the measured variables of the HS variety. Figure 7b shows a similar result. These results are consistent with the report of Lee et al. (2020).

Principal component one describes sample variation, and PC2 successively explains smaller parts of the original variance. This means that the same principal component describes correlated parameters, and a different principal component describes less correlated parameters (Mishra et al., 2013). From Figure 7a and b PCA reveals that all the parameters were clustered into four quadrants. The right‐hand side of the loading plot is occupied by most of the biochemical variables, and among these variables, TPC is the right topmost parameter for both PC1 and PC2 (Figure 7a), whereas half of the proximate parameters are on the left‐hand side of the loading plot (Figure 7a). On the contrary, Figure 7b has all the phenolic compounds and antioxidant activity on the right‐hand side of the loading plot, and only crude fat and protein are on the left‐hand side of the loading plot. Chlorogenic acid recorded the highest positive loading for both PC1 and PC2 on the right upper side of the loading plot (Figure 7b).

In Figure 7a, the first quadrant, consisting of protein, TPC, moisture content, crude fiber, and TPC, exhibits a strong correlation with both PC1 and PC2 positively, whereas the second quadrant, containing FRAP and TPAs, correlates with PC1 positively and with PC2 negatively. The third quadrant, which correlates with PC1 and PC2 negatively, contains CA, total solids, and crude fat. The fourth quadrant, consisting of DPPH radical scavenging activity and ash, shows a high positive loading with PC2 and negative loading with PC1. The results also reveal that the HS variety contained higher TPC and DPPH radical scavenging activity. In Figure 7b, CA, TPC, TPAs, and FRAP make up quadrant one; DPPH radical scavenging activity and TFC make up quadrant two; and only crude protein and fat are in quadrants three and four, respectively. Chlorogenic acid, TPC, TPAs, and FRAP, located in the same quadrant, indicate no differences between the antioxidant mechanisms of these compounds. This interpretation suggests that the HS variety could be considered for its higher TPC, CA, and antioxidant activity and possibly be used for the extraction of CA. A strong negative correlation between TPC and crude protein suggests an interaction. This implies that TPC decreases with increasing crude protein content and vice versa through bonding as stated by Alu’datt et al. (2013). When the protein content of HS is high, more of its polyphenols will be bonded to it. This reduces the quantity of free polyphenols in HS and subsequently decreases the amount of CA in it. A Pearson correlation analysis indicated a similar result (Table S2b).

## 4 CONCLUSION

In the current study, ultrasonic parameters for bioactive CA extraction were optimized to achieve high CA yields, and we evaluated the physicochemical and bioactive properties of the HS variety. The study established an acceptable model for extracting CA with high yields and increased antioxidative activity from soybeans (the heilong48 variety) with a desirability function of 0.918. Scanning electron microscopy results confirmed the structural alterations of the HS variety caused by the optimized ultrasonic parameters. Also, the HS variety significantly contained high TPC, TPAs, and CA as well as high antioxidant properties. A high FRAP with respect to the DPPH free radical scavenging activity of the HS variety was obtained. Also, a protein–phenolic interaction (a negative correlation—a high protein content resulted in low TPC) in the HS variety was observed. The studied sample had low moisture content and so could have an extended shelf life under good storage conditions. It contained high ash content, which signified a good source of minerals and relatively low fat. The use of the HS variety for CA extraction could be economical because it is cheap and available all year round. Further studies should focus on the antimicrobial activity and/or bioactivity of CA from this soybean variety (the heilong48 variety).

Supporting information for this work can be found in the online version of the paper.

## CONFLICT OF INTEREST

The authors declare that they do not have any conflicts of interest.

## AUTHOR CONTRIBUTIONS

Nelson Dzidzorgbe Kwaku Akpabli‐Tsigbe: Conceptualization (equal); data curation (equal); formal analysis (equal); investigation (equal); methodology (equal); software (equal); validation (equal); visualization (equal); writing – original draft (equal); and writing – review and editing (equal). Yongkun Ma: funding acquisition (equal); project administration (equal); resources (equal); and supervision (equal). John‐Nelson Ekumah: Investigation (equal). Juliet Osabutey: Writing – review and editing (equal). Jie Hu: Investigation (equal). Manqing Xu: Investigation (equal). Nana Adwoa Nkuma Johnson: Investigation (equal). Benjamin Kumah Mintah: Writing – review and editing (equal).

## Supporting information

Supplementary Material

Supplementary Material

## Data Availability

The data that support the findings of this study are available from the corresponding author upon reasonable request.
