# Supplementary material for: Ultrasonic‐assisted extraction of bioactive chlorogenic acid from heilong48 soybean variety: Parametric optimization and evaluation of physicochemical and bioactive properties
Source: Food Sci Nutr. 2022 Mar 11;10(4):985–1002. doi: 10.1002/fsn3.2670 (PMC9007310; doi:10.1002/fsn3.2670)
Supplement: Supplementary file 1 — Supplementary Material [file FSN3-10-985-s001.docx]

**Supporting information 1**

**Ultrasonic-assisted extraction of bioactive chlorogenic acid from heilong48 soybean variety: parametric optimization and evaluation of physicochemical and bioactive properties**

**Nelson Dzidzorgbe Kwaku Akpabli-Tsigbe^a,b^, Yongkun Ma^a*^,** **John-Nelson Ekumah^a,b^, Juliet Osabutey^c,d^, Jie Hu^a^, Manqing Xu^a^, Nana Adwoa Nkuma Johnson^a^, Benjamin Kumah Mintah^b^**

^a^School of Food and Biological Engineering, Oversea College of Education, Jiangsu University, 301#, Xuefu Road, Zhenjiang 212013, Jiangsu, P. R. of China.

^b^Department of Nutrition and Food Science, College of Basic and Applied Sciences, University of Ghana, P. O. Box LG 134, Legon-Ghana.

^c^Department of Early Childhood Education, University of Education, P. O. Box 25, Winneba, Ghana.

^d^Virtuous Experimental School, P. O. Box AH 106, Achimota-Accra, Ghana, West Africa.

*Corresponding author: Email: [mayongkun@ujs.edu.cn](mailto:mayongkun@ujs.edu.cn) (Yongkun Ma)

**TABLES**

**Table S1a: Experimental factors and their coding for Box-Behnken Design (BDD)**

|  | **Factors** | **A:** | **B:** | **C:** | **D:** |
| --- | --- | --- | --- | --- | --- |
|  |  | **Frequency (kHz)** | **Power density (W/L)** | **Temperature (^o^C)** | **Time (min)** |
|  | -1 | 20 | 30 | 35 | 25 |
| Coding | 0 | 40 | 40 | 40 | 30 |
|  | +1 | 60 | 50 | 45 | 35 |

**Table S1b. Multiple response optimization and verification of the predictive model.**

| **Name** | **Aim** | **Lower level** | **Upper level** | **Importance** | **Optimum** | **Verification test** | **RE** | **RSE (%)** |
| --- | --- | --- | --- | --- | --- | --- | --- | --- |
| Frequency | is in range | 20 | 60 | 3 | 20.000 | 20.00 |  |  |
| Power density | is in range | 30 | 50 | 3 | 30.000 | 30.00 |  |  |
| Temperature | is in range | 35 | 45 | 3 | 37.935 | 37.90 |  |  |
| Time | is in range | 25 | 35 | 3 | 27.978 | 28.00 |  |  |
| CA yield (mg/g) | maximize | 1.34 | 4.99 | 3 | 4.990±0.079 | 5.007±0.033 | 0.0034 | 0.34 |
| DPPH (μmol AA eq/g dry sample) | maximize | 90.01 | 93.76 | 3 | 93.171±0.369 | 93.197±0.213 | 0.0002 | 0.02 |
| Desirability |  |  |  |  | 0.918 |  |  |  |

RE: Relative Error RSE: Residual Standard Error

**Table S2a. Pearson’s correlation coefficients among total polyphenol content (TPC), total phenolic acids (TPA), chlorogenic acids (CA), total flavonoid content (TFC), and antioxidant activity** **[2,2-diphenyl-1-picrylhydrazyl (DPPH) radical scavenging activity and ferric reducing antioxidant power (FRAP)]**

|  | TFC | TPC | TPA | CA | DPPH |
| --- | --- | --- | --- | --- | --- |
| TPC | 0.500** |  |  |  |  |
| TPA | 0.866** | 0.866** |  |  |  |
| CA | -0.360 | 0.628** | 0.154* |  |  |
| DPPH | 0.922** | 0.796** | 0.992** | 0.029* |  |
| FRAP | 0.856** | 0.876** | 1.000** | 0.175* | 0.989** |

* Correlation was significant at 0.05 level (two-tailed).

** Correlation was significant at 0.01 level (two-tailed).

**Table S2b. Pearson’s correlation coefficients among total polyphenol content (TPC), total phenolic acids (TPA), chlorogenic acids (CA) total flavonoid content (TFC), antioxidant activity (DPPH and FRAP), crude fat and crude protein**

|  | TFC | TPC | TPA | CA | DPPH | FRAP | Crude fat |
| --- | --- | --- | --- | --- | --- | --- | --- |
| TPC | 0.500^*^ |  |  |  |  |  |  |
| TPA | 0.866^**^ | 0.866^**^ |  |  |  |  |  |
| CA | -0.360 | 0.628^**^ | 0.154^*^ |  |  |  |  |
| DPPH | 0.922^**^ | 0.796^**^ | 0.992^**^ | 0.029^*^ |  |  |  |
| FRAP | 0.856^**^ | 0.876^**^ | 1.000^**^ | 0.175^*^ | 0.989^**^ |  |  |
| Crude fat | -1.000 | -0.496 | -0.864 | 0.365^*^ | -0.920 | -0.853 |  |
| Crude protein | -0.500 | -1.000 | -0.866 | -0.628 | -0.796 | -0.876 | 0.496^*^ |

* Correlation was significant at 0.05 level (two-tailed).

** Correlation was significant at 0.01 level (two-tailed).
