# Supplementary material for: Ultrasonic‐assisted extraction of bioactive chlorogenic acid from heilong48 soybean variety: Parametric optimization and evaluation of physicochemical and bioactive properties
Source: Food Sci Nutr. 2022 Mar 11;10(4):985–1002. doi: 10.1002/fsn3.2670 (PMC9007310; doi:10.1002/fsn3.2670)
Supplement: Supplementary file 2 — Supplementary Material [file FSN3-10-985-s002.docx]

**Supporting information 2**

**Ultrasonic-assisted extraction of bioactive chlorogenic acid from heilong48 soybean variety: parametric optimization and evaluation of physicochemical and bioactive properties**

**Nelson Dzidzorgbe Kwaku Akpabli-Tsigbe^a,b^, Yongkun Ma^a*^,** **John-Nelson Ekumah^a,b^, Juliet Osabutey^c,d^, Jie Hu^a^, Manqing Xu^a^, Nana Adwoa Nkuma Johnson^a^, Benjamin Kumah Mintah^b^**

^a^School of Food and Biological Engineering, Oversea College of Education, Jiangsu University, 301#, Xuefu Road, Zhenjiang 212013, Jiangsu, P. R. of China.

^b^Department of Nutrition and Food Science, College of Basic and Applied Sciences, University of Ghana, P. O. Box LG 134, Legon-Ghana.

^c^Department of Early Childhood Education, University of Education, P. O. Box 25, Winneba, Ghana.

^d^Virtuous Experimental School, P. O. Box AH 106, Achimota-Accra, Ghana, West Africa.

*Corresponding author: Email: [mayongkun@ujs.edu.cn](mailto:mayongkun@ujs.edu.cn) (Yongkun Ma)

**FIGURE CAPTIONS**

Figure S1. Perturbation plot for (a) chlorogenic acid yield (b) 2,2-diphenyl-1-picrylhydrazyl (DPPH) radical scavenging activity of ultrasound treated heilong48 soybean sample.

Figure S2. Protein-fat-polyphenol interaction: (a) Correlation (*r^2^* = 1.000) between total polyphenol content (TPC) and crude protein content of heinong48 soybean variety. (b) Correlation *(r^2^* = 0.246) between total polyphenol content (TPC) and crude fat content of heinong48 soybean variety.

| 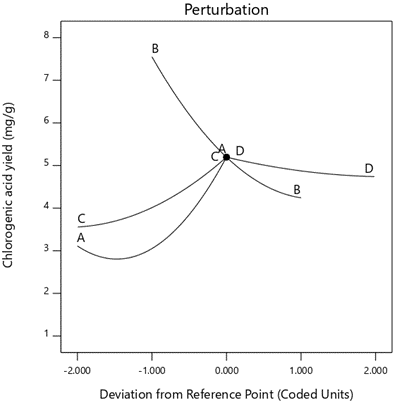  **(a)**  **(b)** | 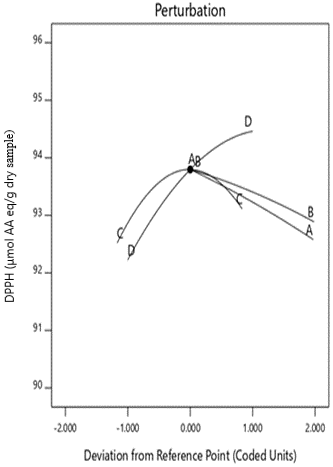 |
| --- | --- |

**Figure S1. Perturbation plot for (a) chlorogenic acid yield (b) 2,2-diphenyl-1-picrylhydrazyl (DPPH) radical scavenging activity of ultrasound treated heilong48 soybean sample.**


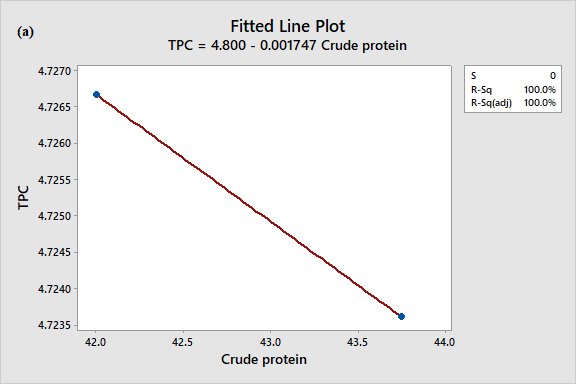


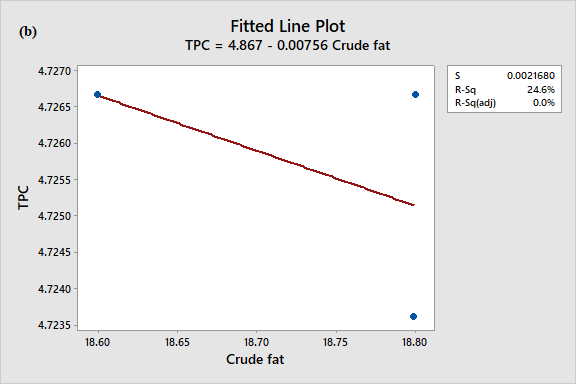


**Figure S2. Protein-fat-polyphenol interaction: (a) Correlation (*r^2^* = 1.000) between total polyphenol content (TPC) and crude protein content of heinong48 soybean variety. (b) Correlation *(r^2^* = 0.246) between total polyphenol content (TPC) and crude fat content of heinong48 soybean variety.**
